# Subclinical gait disturbance and postoperative gait improvement in patients with degenerative cervical myelopathy

**DOI:** 10.1038/s41598-021-90884-2

**Published:** 2021-05-27

**Authors:** Dong-Ho Lee, Jong Yoon Yoo, Jae Hwan Cho, Chang Ju Hwang, Choon Sung Lee, Chunghwan Kim, Jung-Ki Ha, Kun-Bo Park

**Affiliations:** 1 Department of Orthopedic Surgery, Asan Medical Center, University of Ulsan College of Medicine, Seoul, Korea; 2 Department of Rehabilitation Medicine, Asan Medical Center, University of Ulsan College of Medicine, Seoul, Korea; 3 Department of Orthopedic Surgery, Gangneung Asan Hospital, University of Ulsan College of Medicine, Gangneung, Korea; 4 Division of Pediatric Orthopedic Surgery, Severance Children’s Hospital, Yonsei University College of Medicine, 50-1 Yonsei-ro, Seodaemun-gu, Seoul, 03722 Korea

**Keywords:** Diseases, Health care, Signs and symptoms

## Abstract

This study aimed to evaluate the subclinical gait abnormalities and the postoperative gait improvements in patients with degenerative cervical myelopathy using three-dimensional gait analysis. We reviewed the gait analysis of 62 patients who underwent surgical treatment for degenerative cervical myelopathy. The asymptomatic gait group included 30 patients and the gait disturbance group included 32 patients who can walk on their own slowly or need assistive device on stairs. The step width (17.2 cm vs. 15.9 cm, *P* = 0.003), stride length (105.2 cm vs. 109.1 cm, *P* = 0.015), and double-limb support duration (13.4% vs. 11.7%, *P* = 0.027) improved only in the asymptomatic gait group. Preoperatively, the asymptomatic gait group exhibited better maximum knee flexion angle (60.5° vs. 54.8°, *P* = 0.001) and ankle plantarflexion angle at push-off (− 12.2° vs. − 6.5°, *P* = 0.001) compared to the gait disturbance group. Postoperatively, maximum knee flexion angle (62.3° vs. 58.2°, *P* = 0.004) and ankle plantarflexion angle at push-off (− 12.8° vs. − 8.3°, *P* = 0.002) were still better in the asymptomatic gait group, although both parameters improved in the gait disturbance group (*P* = 0.005, 0.039, respectively). Kinematic parameters could improve in patients with gait disturbance. However, temporospatial parameters improvement may be expected when the operative treatment is performed before apparent gait disturbance.

## Introduction

Various degenerative conditions of the cervical spine, including cervical spondylotic myelopathy, degenerative disc disease, and ossification of the posterior longitudinal ligament and ligamentum flavum can result in degenerative cervical myelopathy (DCM)^1^. There are several manifestations of DCM, including clumsy hands, paresthesia, neck pain, and gait disturbances^2^. Gait disturbances in DCM are caused by a spinal cord impairment lesion; the severity of gait disturbance is diverse and should be systematically examined, as they could be misunderstood as a symptom of lumbar spinal disease^3–5^. However, functional scales (GRASSP-M, Nurick, and JOA) designed to evaluate disability in patients with DCM provide only qualitative information of gait disability, rather than quantitative assessment, and may not be sensitive to less pronounced gait changes since a single category often encompasses a wide range of severities or do not include gait abnormality^6–8^.

Three-dimensional gait analysis can provide detailed and quantifiable information about gait parameters. Previous studies suggested slow walking speed or reduced joint range of motion (ROM) as pathologic gait parameters in DCM, and reported an improvement of gait parameters after operation^9–11^. However, the decrease in gait parameters was diverse, and Malone et al*.* found that temporospatial or other kinematic parameters showed no improvement after operation^9,12^. This inconsistent result may be related to the different severity of DCM in terms of gait disturbance. Patients may have only upper extremity symptoms and may not be aware of their gait disability, or the severely affected gait disturbance may not improve after operation^2,13,14^. Identifying quantitative gait differences in patients with DCM would contribute to the detection of subtle preoperative impairments and help predict postoperative improvements.

In this study, we reviewed pre- and postoperative gait analysis of DCM patients who had gait disturbance and those without gait symptoms. The current study aimed to evaluate (1) preoperative subclinical gait abnormalities in patients with DCM and (2) the improvements in gait parameters after surgery using three-dimensional gait analysis in patients with and without apparent gait abnormality.

## Results

### Participants

Between asymptomatic gait and gait disturbance groups, there was no difference in the age at surgery (61.4 ± 10.8 years vs. 55.1 ± 9.0 years, *P* = 0.437), duration of preoperative symptoms (11.5 ± 4.3 months vs. 12.3 ± 5.1 months, *P* = 0.553), and anterior/posterior operation (19/11 vs. 21/11, *P* = 0.53). Both pre- (14.6 ± 1.9 vs. 11.6 ± 2.3) and postoperative (15.1 ± 1.7 vs. 12.4 ± 3.0) JOA scores were higher in the asymptomatic gait group (Table 1). JOA scores improved significantly after surgery in both groups (*P* = 0.013 for asymptomatic gait and *P* = 0.016 for gait disturbance group). However, there was no significant change in lower limb motor function grade according to JOA (*P* = 0.083 for asymptomatic gait and *P* = 0.231 for gait disturbance group).

**Table 1:** Patient characteristics of asymptomatic gait and gait disturbance groups.

Characteristics	Asymptomatic gait	Gait disturbance	*P* value
Age (years)	61.4 ± 10.8	55.1 ± 9.0	0.437
Duration of symptoms before surgery (months)	11.5 ± 4.3	12.3 ± 5.1	0.553
Sex (Male:Female)	22:8	24:8	0.162
Body mass index (kg/m^2^)	27.4 ± 6.5	27.8 ± 10.8	0.860
**JOA scores**			
Preoperative	14.6 ± 1.9	11.6 ± 2.3	0.001*
Postoperative	15.1 ± 1.7	12.4 ± 3.0	0.001*
**Operation**			
Anterior	19	21	0.530
Posterior	11	11	

*JOA* Japanese Orthopedic Association.

*P < 0.05.

### Comparison of temporospatial parameters between groups

Preoperatively, patients in the gait disturbance group exhibited a shorter stride length (105.2 ± 11.5 cm vs. 94.2 ± 23.4 cm, *P* = 0.001) and a slower walking speed (93.9 ± 13.6 cm/s vs. 81.5 ± 24.9 cm/s, *P* = 0.001), in addition to their longer standing phase duration (61.8 ± 4.6% vs. 64.2 ± 5.7%, *P* = 0.001) and double-limb support duration (13.4 ± 3.7% vs. 16.1 ± 7.3%, *P* = 0.011), compared to the asymptomatic gait group. However, there was no difference in the step width (17.2 ± 3.4 cm vs. 17.4 ± 2.4 cm, *P* = 0.553).

Postoperatively, patients in the gait disturbance group continued to have a shorter stride length (109.1 ± 15.2 cm vs. 97.2 ± 23.7 cm, *P* = 0.001) and slower walking speed (96.7 ± 22.1 cm/s vs. 85.3 ± 24.9 cm/s, *P* = 0.008) with longer standing phase duration (61.3 ± 3.2% vs. 63.3 ± 3.9%, *P* = 0.003) and double-limb support duration (11.7 ± 4.3% vs. 14.8 ± 9.2%, *P* = 0.016). Furthermore, postoperative step width became longer compared to the asymptomatic gait group (15.9 ± 3.4 cm vs. 18.7 ± 8.8 cm, *P* = 0.021) (Table 2).

**Table 2:** Comparison of temporospatial parameters between asymptomatic gait and gait disturbance groups.

Parameters	Asymptomatic gait	Gait disturbance	*P* value
**Preoperative**			
Step width (cm)	17.2 ± 3.4	17.4 ± 2.4	0.553
Stride length (cm)	105.2 ± 11.5	94.2 ± 23.4	0.001*
Velocity (cm/s)	93.9 ± 13.6	81.5 ± 24.9	0.001*
Duration of standing phase (% of gait cycle)	61.8 ± 4.6	64.2 ± 5.7	0.001*
Duration of double-limb support (% of gait cycle)	13.4 ± 3.7	16.1 ± 7.3	0.011*
**Postoperative**			
Step width (cm)	15.9 ± 3.4	18.7 ± 8.8	0.021*
Stride length (cm)	109.1 ± 15.2	97.2 ± 23.7	0.001*
Velocity (cm/s)	96.7 ± 22.1	85.3 ± 24.9	0.008*
Duration of standing phase (% of gait cycle)	61.3 ± 3.2	63.3 ± 3.9	0.003*
Duration of double-limb support (% of gait cycle)	11.7 ± 4.3	14.8 ± 9.2	0.016*

*P < 0.05.

### Changes in temporospatial parameters

In the asymptomatic gait group, step width (17.2 ± 3.4 cm vs. 15.9 ± 3.4 cm, *P* = 0.003) and double-limb support duration (13.4 ± 3.7% vs. 11.7 ± 4.3%, *P* = 0.027) decreased after surgery, whereas stride length increased significantly (105.2 ± 11.5 cm vs. 109.1 ± 15.2 cm, *P* = 0.015). There were no changes in velocity (*P* = 0.317) and duration of the standing phase (*P* = 0.552).

However, in the gait disturbance group, temporospatial parameters did not improve postoperatively (*P* = 0.225, 0.119, 0.107, 0.257, and 0.355 for step width, stride length, velocity, duration of the standing phase, and duration of double-limb support duration, respectively).

### Comparison of kinetic and kinematic parameters between groups

Preoperatively, the gait disturbance group had a declined maximum knee flexion angle during the swing phase (60.5 ± 6.4° vs. 54.8 ± 9.5°, *P* = 0.001) and ankle plantarflexion angle at push-off (− 12.2 ± 7.4° vs. − 6.5 ± 6.5°, *P* = 0.001) compared to the asymptomatic gait group (Table 3).

**Table 3:** Comparison of preoperative kinetic and kinematic parameters between groups.

Parameters	Asymptomatic gait	Gait disturbance	*P* value
**Kinematics (°)**			
Mean pelvic anterior tilt angle	9.0 ± 6.0	9.9 ± 8.2	0.493
Maximum hip extension angle	− 4.1 ± 14.5	− 2.9 ± 13.9	0.660
Maximum knee flexion angle	60.5 ± 6.4	54.8 ± 9.5	0.001*
Ankle plantarflexion angle at push-off	− 12.2 ± 7.4	− 6.5 ± 6.5	0.001*
Ankle plantarflexion angle at initial contact	− 3.0 ± 4.3	− 2.6 ± 3.7	0.502
**Kinetics**			
Maximum ankle moment (Nm/kg)	0.9 ± 0.4	0.9 ± 0.4	0.398
Maximum ankle power generation (W/kg)	1.1 ± 0.7	1.3 ± 3.1	0.637

Negative values indicate hip extension and ankle plantarflexion.

*P < 0.05.

After surgery, the gait disturbance group still demonstrated a less maximum knee flexion angle during the swing phase (62.3 ± 5.4° vs. 58.2 ± 9.7°, *P* = 0.004) and ankle plantarflexion angle at push-off (− 12.8 ± 8.2° vs. − 8.3 ± 8.1°, *P* = 0.002) compared to the asymptomatic gait group (Table 4).

**Table 4:** Comparison of postoperative kinetic and kinematic parameters between groups.

Parameters	Asymptomatic gait	Gait disturbance	*P* value
**Kinematics (°)**			
Mean pelvic anterior tilt angle	10.6 ± 6.1	11.6 ± 7.7	0.455
Maximum hip extension angle	− 3.8 ± 8.9	0.7 ± 16.2	0.057
Maximum knee flexion angle	62.3 ± 5.4	58.2 ± 9.7	0.004*
Ankle plantarflexion angle at push-off	− 12.8 ± 8.2	− 8.3 ± 8.1	0.002*
Ankle plantarflexion angle at initial contact	− 3.6 ± 3.9	− 2.7 ± 4.1	0.205
**Kinetics**			
Maximum ankle moment (Nm/kg)	0.9 ± 0.4	0.9 ± 0.3	0.426
Maximum ankle power generation (W/kg)	1.2 ± 6.9	1.1 ± 0.7	0.234

Negative values indicate hip extension and ankle plantarflexion.

*P < 0.05.

### Changes in kinetic and kinematic parameters

In the asymptomatic gait group, there were no significant changes in the mean pelvic anterior tilt (*P *= 0.066), maximum hip extension angle (*P *= 0.625), maximum knee flexion angle (*P *= 0.062), maximum ankle moment (*P *= 0.798), and maximum ankle power generation (*P *= 0.287). However, the ankle plantarflexion angle at push-off increased significantly (− 12.2 ± 7.4° vs. − 12.8 ± 8.2°, *P *= 0.042).

In the gait disturbance group, the mean pelvic anterior tilt (9.9 ± 8.2° vs. 11.6 ± 7.7°, *P *= 0.028) increased and the maximum hip extension angle decreased (− 2.9 ± 13.9° vs. 0.7 ± 16.2°, *P *= 0.006) after surgery. The maximum knee flexion angle (54.8 ± 9.5° vs. 58.2 ± 9.7°, *P *= 0.005) and the ankle plantarflexion angle at push-off (− 6.5 ± 6.5° vs. − 8.3 ± 8.1°, *P *= 0.039) increased significantly. After surgery, the maximum ankle moment increased significantly (0.9 ± 0.4 Nm/kg vs. 0.9 ± 0.3 Nm/kg, *P *= 0.024), whereas the maximum ankle power generation (*P *= 0.583) remained unchanged.

**Figure 1:**
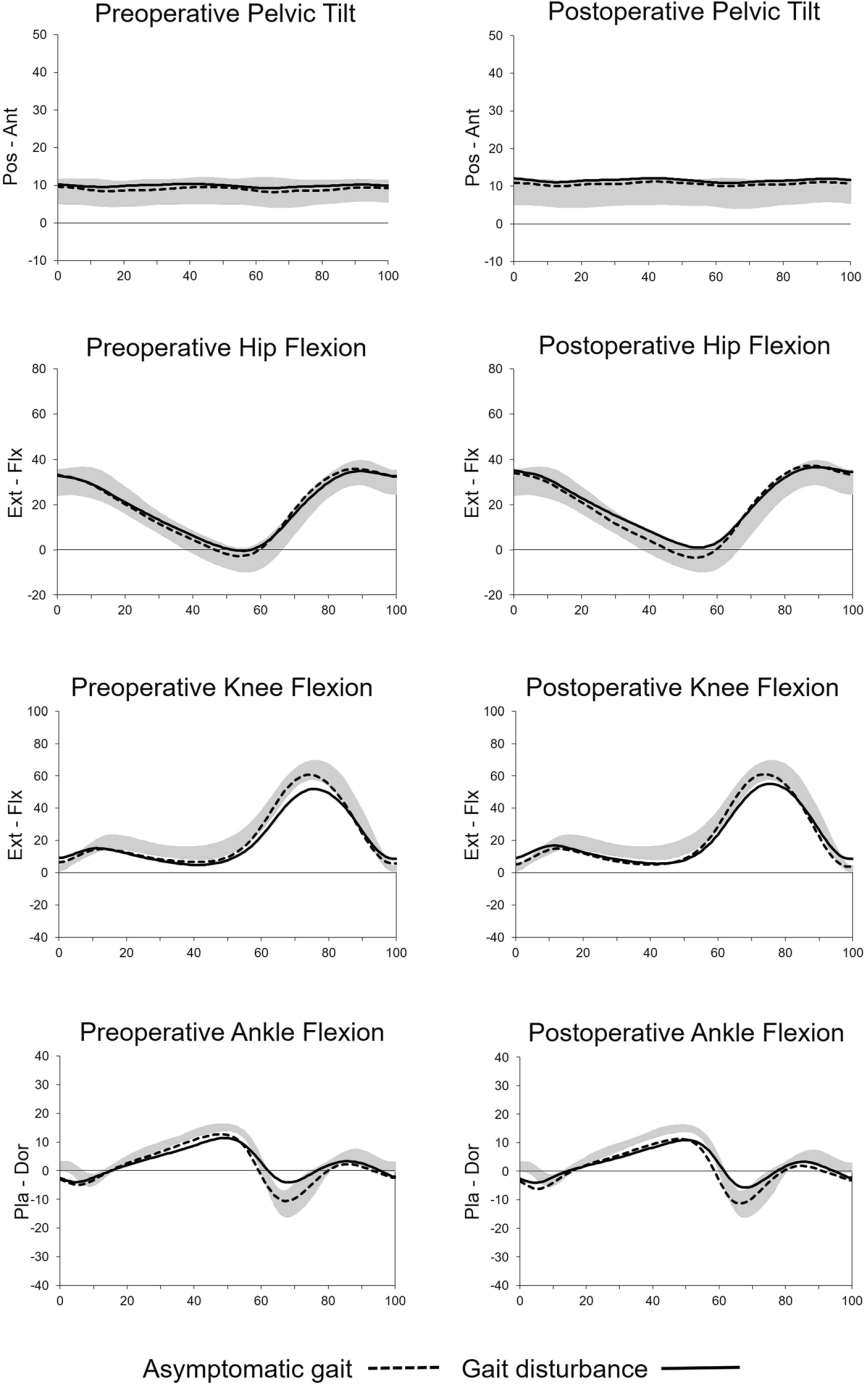
Kinematic graphs of the pelvis, hip, knee, and ankle before and after surgery. Preoperatively, knee flexion during the swing phase and ankle plantarflexion at push-off were decreased in the gait disturbance group compared to the asymptomatic gait group. These factors improved after surgery, but remained lower than in the asymptomatic gait group. In both groups, the ankle angle at initial contact demonstrated plantarflexion both before and after surgery. *Ant* anterior, *Dor* dorsiflexion, *Ext* extension, *Flx* flexion, *Pla* plantarflexion, *Pos* posterior.

In both groups, the ankle plantarflexion at initial contact was noted preoperatively (− 3.0 ± 4.3° in asymptomatic gait group vs. − 2.6 ± 3.7° in gait disturbance group, *P*=0.502) and postoperatively (− 3.6 ± 3.9° in asymptomatic gait group vs. − 2.7 ± 4.1° in gait disturbance group, *P*=0.205) (Figure 1). This ankle plantarflexion at initial contact did not change in both the asymptomatic gait (*P*=0.377) and the gait disturbance (*P*=0.851) groups by operation.

## Discussion

The natural progression of DCM is variable, but typically manifests as a slow, stepwise worsening of symptoms^15^. Previous studies have addressed the importance of early surgery to obtain substantial postoperative improvement^2,13^. Early diagnosis is difficult, as DCM is often painless at onset; as a result, DCM may not be diagnosed until it progresses to a late stage. Myelopathic gait is one of the DCM symptoms^16^, but many patients do not also exhibit a definite gait abnormality. Radcliff et al.^14^ found that 18% of patients with hip fracture manifested clinical findings consistent with DCM. Other studies reported an association between myelopathic gait and an increased risk of falling or subsequent hip (and other) fragility fractures^17,18^. Early detection of gait disturbances in DCM is important, not only for better prognosis of DCM after surgery, but also to prevent fractures related to the gait disturbance and to maintain the quality of life. In this study, we evaluated the subclinical myelopathic gait preoperatively and the improvements of gait abnormalities after surgery.

Previous studies reported reduced ankle plantarflexion and knee ROM as pathologic changes in kinematics, with decrease in gait speed and stride length and increase in double-limb support time and step width^4,9,19–22^. The gait disturbance group in this study also showed less knee and ankle ROM with a declined temporospatial parameters compared to the asymptomatic gait group. However, the decrease in knee flexion was not definite in the asymptomatic gait group, and the maximum knee flexion did not increase postoperatively. Instead, step width and double-limb support duration decreased, and stride length increased with the increase of ankle plantarflexion at push-off after surgery only in the asymptomatic gait group. Nagai et al.^23^ proposed gait speed and stride length as indices for evaluating progressive gait abnormalities. Considering the gait parameter improvement in the asymptomatic gait group, an increase of step width and double-limb support duration and a reduced stride length may be early signs of DCM. However, reduced gait speed or stride length may also reflect a patient’s comfortable gait speed, which may be influenced by aging or the patient’s preference. Comparing the gait patterns of people with untreated DCM to those of age- and gender-matched healthy controls, Malone et al.^5^ found that key differences exist in the motor strategies used in the terminal stance phase of gait (including peak ankle plantarflexion), which cannot be explained by speed alone. An increase of step width and double-limb support duration and a decrease in ankle push-off at terminal stance indicate early gait deterioration in patients with DCM.

Another abnormal kinematic finding in the asymptomatic gait group was ankle plantarflexion at initial contact. The ankle plantarflexion at initial contact was noted in both groups pre- and postoperatively. This abnormal ankle plantarflexion may be misdiagnosed as a foot drop that could be seen in patients with lumbar spinal disease. However, patients with foot drop due to weakness in ankle dorsiflexor walk with increased knee flexion for foot clearance. In patients with DCM, preoperative knee flexion was decreased, postoperative gait analysis showed improved knee flexion and an increase in stable foot clearance with a more powerful push-off demonstrating smooth and coordinated movement. Myelopathic gait is classically described as a “spastic pattern gait” and is accompanied by hyperreflexia and incoordination of agonist/antagonist muscles^12,24^. However, Malone et al.^24^ concluded the hyper-excitability of the stretch reflex did not contribute to the abnormal kinetic and kinematics. They suggested that paresis and poor proprioception were associated with gait impairment. Paresis may be related to the decrease of ankle plantarflexion at push-off. Proprioception plays an important role in smooth, coordinated activation of the extremities. Sensory information for proprioception is collected at the nerve endings and neural impulses subsequently travel toward the cerebral cortex via the posterior columns of the spinal cord^22,25^. Any disorder within these neural pathways, such as DCM, can impair proprioception. This may manifest as reduced knee flexion during swing phase known as spastic gait. In our opinion, ankle plantarflexion at initial contact may be another early sign of spastic gait related to the impaired proprioception.

Improvement in walking speed, step length, and knee ROM have been reported after DCM surgery^11,26^. Although we observed significant improvements in temporospatial parameters in the asymptomatic gait group, the gait disturbance group exhibited no significant improvements in these temporospatial parameters, despite improvements in kinetic and kinematic parameters. Furthermore, step width improved only in the asymptomatic gait group, which was relatively worse in the gait disturbance group postoperatively, although there was no difference preoperatively. The importance of early decompressive surgery has been previously discussed^8,12,13^. Malone et al.^12^ found no significant changes in temporospatial parameters at 1 year after surgery for DCM. Garza-Ramos et al.^8^ concluded that patients with a higher preoperative Nurick grade with symptoms for more than 12 months may have significantly lower odds of gait improvement after surgery. In our gait disturbance group, the average duration of symptoms before surgery was 12.3 months. Furthermore, postoperative parameters in the gait disturbance group remained worse than those in the asymptomatic gait group. Early surgery appears mandatory for recovery of gait impairment.

Several qualitative functional outcome scales (e.g., JOA score, Nurick grade, European Myelopathy Score, and Myelopathy Disability Index scale) have been used clinically to assess the results of DCM surgery. However, in addition to having low sensitivity, these outcome scales are subjective, categorical, and overly simplified. In this study, lower limb motor function grade according to JOA did not demonstrate a significant change, even though the temporospatial parameters in the asymptomatic gait group and kinematic parameters in the gait disturbance group were improved. These results suggest that the quantitative assessment of spasticity and deterioration of proprioception, based on gait analysis, could play an important role in early detection of gait disturbances in patients with DCM, as well as in the assessment of postoperative improvements. Decompressive surgery after early detection of DCM is expected to reduce complications associated with gait disturbances, such as fragility fractures, and improve the patients’ quality of life.

This study had several limitations. First, it had a retrospective design, and the operative methods differed between anterior and posterior decompression procedures. However, the surgical approach and the duration of symptoms were not significantly different between the asymptomatic gait and gait disturbance groups. Second, we only selected patients who could walk at least 20 m on their own; therefore, patients with severe gait disturbance or those who could only walk with an assistive device were not evaluated. Even when the patients are non-ambulatory, improved walking ability may be observed after surgery^13^. In this study, there was no significant improvement in the temporospatial parameters of the gait disturbance group; therefore, other study protocol besides the gait analysis should be added for the severely affected patients in the future. Finally, we set the end-point of postoperative improvement at 6 months considering the presence of other degenerative diseases, such as osteoarthritis or spinal stenosis, in this elderly population. This follow-up period was also based on previous studies ^26,27^. A prospective study involving a larger number of patients (including non-ambulatory patients) and a longer follow-up period would be helpful to further understand the potential improvements in myelopathic gait after DCM surgery.

In conclusion, reduced ankle plantarflexion angle at push-off, increased step width, and increased double-limb support duration may be early signs of DCM. Ankle plantarflexion at initial contact may also indicate early gait deterioration in patients with DCM. Even the patients without gait disability can be benefited from surgery in terms of the temporospatial parameter improvement. In the gait disturbance group, knee ROM and ankle plantarflexion at push-off improved after surgery, but were still lower compared to the asymptomatic gait group. Furthermore, preoperatively declined temporospatial parameters did not improve. Early diagnosis and surgery for DCM may be essential for preserving gait function and improving gait disability.

## Methods

### Study design and materials

This retrospective study was approved by the Asan Medical Center Institutional Review Board, and was conducted in accordance with the Declaration of Helsinki. Informed consent waiver was obtained from the Asan Medical Center Institutional Review Board (2020–1030). We reviewed the patients who had undergone decompressive surgery for DCM by a single surgeon (D.H.L) between 2014 and 2016. The following inclusion criteria were applied in this study: (1) aged 18 years and older, (2) clinical and radiological evidence of DCM, (3) able to walk at least 20 m without assistance from another person or a walking aid, and (4) availability of complete preoperative and postoperative three-dimensional gait analysis. Patients were excluded if they had thoracic or lumbar spinal disease, as well as other conditions that may affect their walking ability (e.g., leg length discrepancy more than 2 cm, degenerative or rheumatoid osteoarthritis, peripheral nerve disease or injury, stiff joint, cardiac disease, respiratory disease, stroke, traumatic brain injury, and myelitis), or had a history of orthopedic surgery or neurosurgery that could affect gait. Sixty-two patients (46 males and 16 females) with a mean age of 58.6 years (range 42 to 76 years) were included in this study.

Since gait analysis was impossible to determine for patients who could not walk independently, all of the included patients had their lower limb motor function graded as 2, 3, or 4, according to the Japanese Orthopedic Association (JOA). Patients with grade 4 (“normal”) were classified as the asymptomatic gait group (n = 30), and those with grade 3 [“possible to walk without cane or aid, but slow” (n = 17)] or grade 2 [“need cane or aid only on stairs” (n = 15)] were combined and classified as the gait disturbance group (n = 32).

### Gait analysis protocol

Gait analysis was performed on a flat-ground, 20-m track using a computerized three-dimensional gait analysis system consisting of six infrared cameras (Motion Analysis®, Santa Rosa, CA, USA). Fifteen retroreflective markers were attached to anatomical landmarks using the Modified Helen Hayes method. The capture volume of the cameras was calibrated prior to each assessment to achieve calibration residuals less than 2.5 mm. Anthropometric data were collected according to a standard protocol. After a warm-up trial, individuals walked continuously and freely along the track five times. Participants were permitted to rest between trials to avoid fatigue. Two force-plates, located under the path, recorded ground reaction forces during walking, and joint moments were expressed as internal moments to counter the ground reaction force. Kinematic data was shown as the angle between two segment axes projected into the each sagittal, coronal, and transverse plane. The gait cycle events (heel strike and toe off) were identified automatically from the force plate data during walking. The kinematic and kinetic data were derived for pelvis, hip, knee, and ankle in three planes.

The following parameters from kinematic and kinetic data were analyzed: temporospatial parameters (step width, stride length, velocity, standing phase duration, and double-limb support duration), kinematic parameters (mean pelvic anterior tilt, maximum hip extension angle, maximum knee flexion angle, ankle plantarflexion angle at push-off, and ankle plantarflexion angle at initial contact), and kinetic parameters (maximum ankle moment and maximum ankle power generation).

Gait analysis was performed before surgery and at 6 months after surgery. This postoperative period was a compromise between increasing the likelihood of optimal surgical recovery and reducing the potential confounding effects of aging and degenerative joint disease during follow-up^27^.

### Statistical analysis

Statistical analysis was performed using the SPSS version 23 (IBM Corp, Armonk, NY). Independent t-tests were used to compare the parameters between asymptomatic gait and gait disturbance groups, and paired t-tests were used to compare the parameters between preoperative and postoperative evaluations. Values are presented as mean and ranges. The statistical significance level was set at *P* < 0.05.

### Ethical approval

This study was approved by the Asan Medical Center Institutional Review Board (IRB No. 2020-1030).

## Data Availability

The datasets generated during and/or analyzed in the current study are available from the corresponding author upon reasonable request.
